# Promoting Family Involvement in the Management of Delirium in Intensive Care: Scoping Review

**DOI:** 10.3390/medicina60121934

**Published:** 2024-11-24

**Authors:** Sandra Lange, Wioletta Mędrzycka-Dąbrowska

**Affiliations:** 1 Department of Internal and Pediatric Nursing, Faculty of Health Sciences, Medical University of Gdańsk, Dębinki 7, 80-211 Gdańsk, Poland; sandra.lange@gumed.edu.pl; 2 Department of Anaesthesiology Nursing & Intensive Care, Faculty of Health Sciences, Medical University of Gdansk, Dębinki 7, 80-211 Gdańsk, Poland

**Keywords:** critical care, intensive care units, delirium management, family involvement, family-centered care, scoping review

## Abstract

*Background*: In recent years, family involvement in ICU patient care has become increasingly significant. Family involvement in delirium management, while desirable, can be difficult for loved ones. Therefore, every attempt should be made and interventions developed to promote and support the family in this process. The aim of this review was to analyze the available literature on interventions and strategies used by ICU staff to support and promote family involvement in the management of delirium in critically ill patients. *Methods*: The databases searched included the following: MEDLINE, CINAHL, and the Cochrane Library. Studies were included in the review if they were conducted in adult intensive care units and/or addressed interventions to support and promote family/relatives’ involvement in delirium management. *Findings*: A total of 368 database articles were reviewed. After removing duplicates and checking for inclusion and exclusion criteria, four articles were finally included in the analysis. Research gaps and content analysis identified promotional and supportive interventions for family involvement in delirium management: (I) Education; (II) Mentoring; (III) Partnership. *Conclusions*: Research gaps to be filled are as follows: (I) the scope of interventions that support and promote family involvement in delirium management; (II) interventions that enhance feelings of efficacy among family members and reduce symptoms of anxiety and depression; and (iii) the impact of specific interventions on patients’ delirium outcomes.

## 1. Background

In recent years, family involvement in intensive care unit (ICU) patient care has become increasingly significant [1,2]. Interventions based on the patient- and family-centered care (PFCC) model have been shown to have a positive impact on both patients and their loved ones [3]. Including them in care can also prevent and shorten the duration of delirium, which is an adverse complication of ICU hospitalization [4,5]. Non-pharmacological interventions are used as the standard of care for delirium prevention and treatment, primarily aimed at minimizing potential delirium triggers [6]. A growing number of studies show the effectiveness of involving the family in the prevention and treatment of delirium by using interventions such as assessment of delirium with scales, flexible visits, cognitive stimulation, and orientation [4,7,8,9]. On the other hand, family involvement can have adverse consequences for family mental health [10]. Therefore, supporting the family and promoting their involvement in delirium management is an important aspect that requires the implementation of proven strategies.

The scoping review aims to examine the available literature on interventions and strategies used by ICU staff that support and promote family involvement in the management of delirium in critically ill patients. The review will identify gaps in existing research and identify future directions for research on optimizing family involvement in delirium patient care.

## 2. Methods

### 2.1. Study Design

A scoping review method was chosen to map concepts relevant to the topic of interventions that promote and support family involvement in delirium management, as well as to identify gaps in the existing literature and provide directions for future research [11]. Scoping reviews rely on evidence from a variety of research methodologies and may also include evidence from non-research sources [12]. The scoping review was conducted in accordance with the methods described in the Joanna Briggs Institute methodology manual for scoping reviews [11] and using the recommendations of the Preferred Reporting Items for Systematic Reviews and Meta-analysis for Scoping Reviews (PRISMA-ScR) guidelines [13]. The Arksey and O’Malley framework was used. This framework consists of five steps: (I) identifying the research questions, (II) identifying relevant studies, (III) selecting studies, (IV) charting the data, and (V) collating, summarizing, and reporting results [14].

### 2.2. Identifying the Research Question

To identify important aspects related to interventions and strategies used by ICU staff that support and promote family involvement in the management of delirium in ICU patients, we developed research questions that clearly define the population, concept, and context (PCC) of the scoping review:

1. What interventions do ICU staff use to promote and support family involvement in delirium management?
2. Are the interventions effective, and what clinical significance do they have?

#### 2.2.1. Population

Studies that were conducted in patient populations in adult intensive care units were included in the review.

We defined an intensive care unit as a hospital ward that provides intensive care to critically ill patients with life-threatening injuries and illnesses.

In this scoping review, adults were defined as those who were 18 years of age or older.

#### 2.2.2. Concept

The subjects of interest were the interventions that ICU medical staff use to support and promote family involvement in the management of delirium in the ICU.

Family was defined as individuals who are related to patients by blood or marriage, and caregivers/relatives were defined as individuals who accompanied patients during their stay in the ICU.

#### 2.2.3. Context

The interventions implemented to support the family in involving them in delirium management, e.g., educational programs, talking to medical staff, educational videos. However, the review did not include interventions in which the family is involved to prevent and reduce the duration of delirium, such as flexible visiting hours and delirium assessment.

### 2.3. Identifying Relevant Studies

Two authors systematically searched the following databases: MEDLINE, CINAHL, and Cochrane Library. These databases were used to access a wide range of English-language literature published between 2017 and 2024 in peer-reviewed journals. The search strategy is presented in Table 1. In addition, the researchers checked the reference list of existing literature reviews to identify eligible articles that may have been missed during the search. The initial search was from inception to May 2024, with a final search in September 2024.

### 2.4. Study Selection

In the first stage, selection was made by reading the titles and abstracts of articles to identify duplicates and exclude ineligible articles. Inclusion and exclusion criteria were in accordance with the PCC. Inclusion criteria for studies in the review were as follows: studies that took place in an adult intensive care unit; involved interventions that promote or support family/relative involvement in delirium management. Articles that were commentaries or editorials, were conducted in the neonatal intensive care unit (NICU), and dealt with family involvement in delirium prevention interventions were excluded from the study. The selection of articles was limited to English-language articles conducted between 2017 and 2024. Any discrepancies were resolved through discussion with the researchers, and, at the end of the selection process, full agreement was reached on the articles to be included.

### 2.5. Charting the Data

Articles were mapped, extracting relevant information such as author, country, study design, setting, study participants, and study purpose (Table 2).

### 2.6. Collating, Summarizing, and Reporting the Results

A narrative synthesis was used to analyze the results of the included studies [20]. The effectiveness of the intervention was analyzed by its effect on outcomes measured in caregivers and the effect size by analyzing whether the interventions were relevant to clinical practice.

The coding process was carried out in two steps. First, qualitative content analysis was used to summarize and synthesize the data from the studies included in the review. Next, conventional content analysis was used, in which codes are derived from the data during analysis. That is, codes are defined during data analysis and derived from the data results. This approach made it possible to highlight general trends in the literature relating to the topic of promoting and supporting family involvement in ICU delirium management [21].

## 3. Results

### 3.1. Study Selection

**Figure 1:**
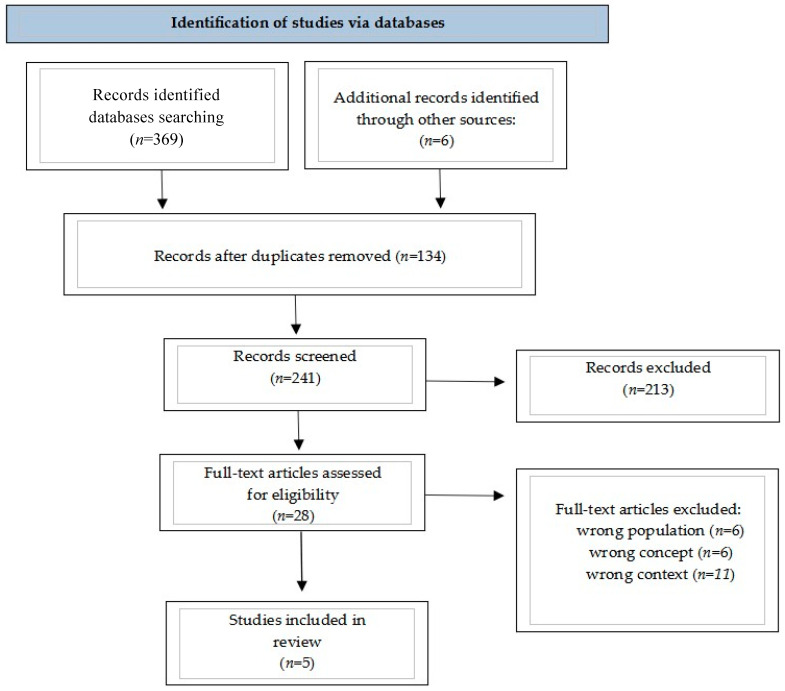
PRISMA flow diagram [22].

The database search resulted in a total of 375 articles. A total of 134 duplicates and 213 ineligible articles were removed from the abstract review. After the full-text analysis of articles, 28 studies were removed due to poor population, concept, and context. A total of 5 studies were included in the analysis (Figure 1).

### 3.2. Study Characteristics

All 5 included studies are quantitative studies. The research designs used are the quasi-experimental study, the intervention study, and the randomized pilot study and nonequivalent control group pretest–posttest nonsynchronized design. In terms of geographic distribution, the studies were conducted in Canada (n = 3) [15,16,17], the USA (n = 1) [19], and in Korea [18]. The interventions were conducted in clinical settings in ICUs with different profiles (oncology ICU, cardiac surgery ICU, general ICU, and surgical ICU) for the family caregivers of ICU patients.

### 3.3. Interventions to Support and Promote Family Involvement in Delirium Management

Three main interventions were identified to support and promote family involvement in delirium management in the ICU: (I) Education; (II) Partnership; (III) Mentoring. A synthesis of the included articles is presented in Table 3.

### 3.4. Education

“Education” is one intervention that has been studied in the literature. Two studies have used family education interventions about delirium in ICU patients [17,18,19].

The studies by Krewulak et al. and Wheeler et al. to measure families’ knowledge of delirium before and after implementation of an educational intervention were used; these involved the caregiver delirium knowledge questionnaire [17,19]. In the Hee et al. study, family education was part of an eight-element multi-component intervention (Program for Preventing Delirium in Geriatric Patients in the Intensive Care Unit). However, this study did not measure caregiver knowledge, either before or after education [18].

### 3.5. Partnership

Another approach to family empowerment in the care of ICU patients with delirium was partnership, which was used in the study by Wheeler et al. In this study, caregivers not only received education about delirium but were also included in the development of the educational video. This approach demonstrates both the potential for partnership between staff and families but also allows the family to find a sense of self-efficacy in caring for their loved one. During the development of the delirium education video, a partnership was formed with, among others, the hospital’s Patient Education Committee, whose members included patients and their caregivers. The idea behind the committee is to ensure that the voices of patients and families are heard and incorporated into hospital projects [19].

### 3.6. Mentoring

A third intervention reported in the literature was mentoring delivered by ICU nurses. The feasibility of this intervention was initially studied in a pilot study and then developed in an intervention study [15,16]. The creation of the intervention followed three steps: (1) develop an understanding of the problem under study; (2) define the objectives of the intervention and identify a theoretical framework to outline the potential mechanisms of action for the intervention; (3) operationalize the intervention and identify its anticipated outcomes. Step one involved conducting a narrative review that increased the understanding of delirium risk factors and outcomes, and identified the goals of interventions conducted with families in the context of delirium. In addition, a research project was conducted to assess the needs of families of patients who have developed delirium, and a list of potential intervention targets was then provided to clinicians and an expert panel. In step two, a theoretical framework was identified that could recommend strategies to increase both the family’s presence at the patient’s bedside and their involvement in the non-pharmacological treatment of delirium. The theoretical framework was selected through a review of nursing theory and a literature search. The final step was to operationalize and identify the expected outcomes by searching the databases again to include any nursing interventions that used Bandura’s principles to increase self-efficacy among adult hospitalized patients. Three objectives were identified for MENTOR_D: (1) to decrease the severity of the manifestations of delirium; (2) to improve patient outcomes (decrease complications, length of stay, and improve recovery); and (3) to improve family outcomes (decrease anxiety, increase self-efficacy). These goals were intended to be achieved by increasing the presence of families at the bedside and their involvement in the non-pharmacological treatment of delirium [16].

## 4. Discussion

This scoping review aimed to examine the available literature on interventions and strategies used by ICU staff to support and promote family involvement in the management of delirium in critically ill patients. Interventions were conducted in clinical settings in ICUs with different profiles (oncology ICU, cardiac surgery ICU, general ICU, and surgical ICU) for family caregivers. Only five studies were identified, two of which were a quasi-experimental study (one pilot study), one was a pilot study that was later developed as an intervention study, and one was a nonequivalent control group pretest-posttest nonsynchronized study. Three types of interventions were identified; these were as follows: (1) Education; (2) Partnership; (3) Mentoring.

The unknown environment of the ICU and the severity of a patient’s condition make family involvement in the care of their loved ones a challenge, often accompanied by fear, anxiety, and suffering [24,25]. Therefore, family involvement in the management of delirium becomes a challenge for both the family and ICU staff [26,27,28]. Family involvement has a significant effect on the incidence of delirium. Family participation has been shown to reduce the incidence of delirium in ICU patients [29]. Moreover, the Michell et al. study confirmed that interventions delivered by the family, such as memory clues or orientation (family photographs, orientation to surroundings), therapeutic or cognitive stimulation (reminiscing, discussing family life), and sensory checks (vision with glasses and hearing with hearing aids), are feasible, as well as acceptable, to both family members and nursing staff [30]. However, this requires adequate training of relatives about delirium, who could then collaborate with medical personnel in the early recognition of the symptoms of delirium. An educational intervention directed at relatives of the elderly about delirium has shown effectiveness in several aspects for both family caregivers and the elderly. First, family caregivers’ knowledge of delirium increased; it was noted that the incidence of delirium in the elderly decreased; and there was a demonstrated effect on reducing stress after receiving education [31]. As families are the people who know their loved ones the most, they are able to identify subtle changes in their relative’s behavior that may be symptoms of delirium [32]. The impact of the intervention on psychological outcomes is equally important, as the prevalence of anxiety symptoms, depression, and post-traumatic stress disorder (PTSD) has been shown to be higher in families of patients diagnosed with delirium while in the ICU [33]. In addition, caregivers of critically ill patients with delirium who have been involved in delirium detection experience anxiety [34]. It is important to understand what the family’s needs and perceptions are in relation to their involvement in caregiving. A qualitative systematic review found that caregivers’ contributions are supported when their knowledge is respected, they are given information, permission, and support to enable the partnership, and communication with staff is clear. Above and beyond this, time spent supporting caregiver education and capacity building in the early stages of admission may be a promising intervention [35]. These tips can be helpful in creating interventions.

### 4.1. Education

Educational interventions were based on a video that addressed aspects such as delirium risk factors, symptoms, and non-pharmacological interventions provided by families. The education was also supplemented with case studies that required the application of the knowledge gained. Although the level of knowledge has not been shown to correlate with anxiety symptoms, studies have indicated the feasibility of this intervention and its acceptability by families. Family knowledge of delirium improved after the intervention. Most families found the educational video helpful, and they used the information in it in caring for their loved ones. Moreover, the study indicated that this intervention could also be implemented virtually. This provides greater flexibility in education, adjusting the time and pace of education to individual needs, and will allow more caregivers to be included in the education. Additionally, it is feasible to implement in emergency situations such as a pandemic [17,19]. In one study conducted on an elderly unit, an educational brochure was developed for educational purposes. The purpose of the delirium brochure was to provide family caregivers with education about delirium and to enable them to be an integral part of the healthcare team. The content of the brochure included helping families understand the difference between delirium and dementia, the signs, symptoms, and causes of delirium, and strategies family members can use to prevent delirium. The initiative provided easy-to-receive education for family caregivers with a recommendation for continued use by nursing staff in targeted settings. Although the analysis showed a willingness to use the brochure in the future, its ongoing dissemination by nursing staff was lacking [36]. The results of an integrative review highlighted that delirium-related educational interventions for caregivers in various hospital units positively affect both caregivers and patient outcomes [37]. In creating educational interventions, researchers should consider how to deliver the content. The form of in-person education is acceptable and feasible; however, it does not provide as much flexibility as virtual education. Adapting the form to meet the needs of family caregivers can influence reaching a wider population. It should be noted that the educational interventions not only improved caregivers’ knowledge of delirium but also resulted in family involvement in delirium management by implementing the non-pharmacological interventions learned during the video to prevent delirium.

### 4.2. Partnership

Strengthening the role of the family can be implemented on a partnership and collaborative basis as early as the development of educational programs, so that the family’s voice is heard and their needs result from a direct narrative with them. In addition, the use of this collaborative model can help to foster a sense of responsibility for their care, as well as strengthen their sense of self-efficacy, although this aspect needs to be studied. Christina Aggar et al., in collaboration with caregivers, developed the web-based Delirium Toolkit to support their integration as partners in the care of hospitalized older adults at risk of delirium by integrating them in the prevention, identification, and management of delirium, as well as ensuring their well-being by reducing caregiver burden and psychological stress and improving satisfaction with care. To do this, they used the eDelphi method, which includes a systematic communication technique, to achieve group consensus [38]. A pre- and post-test intervention study evaluated the feasibility of the Prevention and Early Delirium Identification Carer Toolkit (PREDICT) to support a partnership between caregivers and nurses in the prevention and treatment of delirium. Nurses implemented PREDICT to provide caregivers with information about delirium and strategies for dealing with stress and the burden of caregiving. Results showed that caregivers’ knowledge of delirium increased significantly from admission to discharge from the hospital. In addition, belief in partnership in delirium prevention and management increased among caregivers after the intervention, as did caregivers’ actual use of PREDICT. In contrast, caregiver burden and distress scores did not change significantly from admission to discharge [39]. Similar results were achieved with the implementation of The Nurse/Family Caregiver Partnership for Delirium Prevention (NFCPM), an innovative program that simultaneously teaches family caregivers and nurses about delirium and partnership in prevention. The results of this study showed its feasibility and a significant increase in knowledge about delirium and attitudes toward the intervention group partnership. The NFCPM program addresses the educational and communication needs of both nurses and family caregivers in delirium prevention while enabling nurses and family caregivers to become better informed and more confident in their ability to take action [40]. An integrative literature review whose population was acute care patients showed that collaborative enhancement of caregivers’ and nurses’ knowledge of delirium, combined with education on developing a therapeutic nurse–caregiver relationship, is important for effective partnership in the management of delirium. Supporting an effective partnership is good communication that allows both nurses and caregivers to express their needs [41].

While a partnership approach to promoting and involving the family in delirium management is feasible and beneficial by increasing the family’s active participation in the patient’s care, it does not guarantee a beneficial effect on the caregivers’ mental health. Therefore, further research on interventions should also consider this aspect. Given that studies have shown inadequate knowledge among ICU nurses about delirium and lack of confidence in their delirium detection skills [42], a partnership approach can also be used as joint participation in educational programs.

### 4.3. Mentoring

A key aspect of increasing family involvement in delirium management is to teach them a new role in which they feel comfortable and confident. This process can be supported by mentoring provided by the ICU nurse. This approach requires the nurse (mentor) to skillfully interact with the family. In the Mailhot et al. study, three theories were used to do this. Watson’s human caring theory, which describes a caring human-to-human relationship and is susceptible to promoting the fullness and healing of the family caregiver. Therefore, it was hypothesized that such a relationship could promote caregivers’ learning of new roles. The caring relationship can be implemented through mentoring, as described by Anderson and Shannon, on the basis of an interpersonal relationship between an experienced person—the nurse—and novices—the family. The mentor’s function would be to teach, sponsor, encourage, counsel, and befriend. Bandura’s social cognitive theory was then used to explain how a mentoring relationship could increase a family’s confidence in their ability to perform their new role [16]. Results from this pilot study showed promise in improving patient and family outcomes [15]. The literature describes a successful mentoring relationship experience focused on the learning of health knowledge by caregivers of chronically ill people. The program was associated with a reduction in the caregiving burden on participants and the establishment of a strong bond and relationship between mentor and mentee. Mentoring involved making a significant contribution to the process of mutual teaching and learning through peer co-creation and collaboration [43]. In a study conducted among parents of children with chronic illnesses, peer mentoring has been shown to be perceived as positive and to have a beneficial effect on families, as it provides a sense of being understood and strengthened and reduces anxiety and feelings of isolation [44,45]. Mentoring delivered by an ICU nurse seems to be a promising strategy to support and promote family involvement in delirium management. It involves a broader aspect than just educating the family about delirium. Among other things, it allows for the establishment of a bond between the nurse and the family, which fosters the learning process, strengthening the relationship, motivating, and advising. Finally, it can lead to the strengthening of the family’s function and its participation in delirium management, which will result in improved patient outcomes for delirium. Moreover, the new function of the mentor can become a steppingstone for the development of nurses in this field.

### 4.4. Research Gaps

Research gaps that need to be filled include several aspects. First, the scope of interventions that support and promote family involvement in delirium management. The number of studies that focus on the above interventions is small. Although results have shown the feasibility of the interventions and their positive impact on patients’ families, further research is needed to confirm their effectiveness (in particular, a randomized controlled trial). Second, research should focus on the impact of the interventions used on family psychological outcomes. This could include not only feelings of anxiety and depression but also, for example, self-efficacy and increased satisfaction with ICU care. Third, future research should be directed at assessing how the implementation of particular interventions will directly affect patient outcomes in terms of delirium (i.e., reducing the incidence and duration of delirium)

## 5. Limitations

This review has several limitations that must be taken into account when interpreting it. First, a small number of studies were included in the review. Second, two pilot studies were included in the review. While these show promising results, they may ultimately differ in the final studies. The different methodological approach may negatively affect the generalizability of the results. Third, only literature in English was included in the review, which may have limited the search results.

## 6. Conclusions

In recent years, the role of the family in the ICU has strengthened. There is a greater emphasis on patient- and family-centered care, the key tenets of which include respect for the individual, open staff–family/patient communication, and their active participation, involvement, and cooperation. The positive impact of family involvement in the active care of ICU patients has also been demonstrated in the context of delirium. Family involvement in delirium management, while desirable, can be difficult for relatives. Many data show what non-pharmacological interventions the family can be involved in to prevent and shorten the duration of delirium, but the current literature does very little to specify what strategies should be adopted to get families to cooperate and strengthen their commitment and self-efficacy while providing support, motivation, and well-being.

Therefore, there is a need to develop nursing interventions that support family involvement and minimize the consequences of delirium for both families and patients. Good-quality research is needed to confirm the impact of these interventions on family and patient outcomes. Future studies are needed and should be focused on the following: (I) the scope of interventions that support and promote family involvement in delirium management; (II) interventions that enhance feelings of efficacy among family members and reduce symptoms of anxiety and depression; and (III) the impact of specific interventions on patients’ delirium outcomes.

## 7. Implication for Clinical Practice

Family education interventions are feasible and helpful in involving family members in delirium management. Educational materials should include both theoretical content about delirium (definitions, risk factors, symptoms) and practical tips that the family can use at the patient’s bedside (non-pharmacological interventions to prevent delirium). It may also be helpful to develop case reports as training for the family. To increase family participation in educational interventions, it can be conducted virtually. Educational training on delirium should also be provided to ICU bedside nurses. Education can be provided simultaneously for family and staff on a partnership basis. Implementing interventions can be a challenge in the face of staffing deficiencies that are not uncommon and against the large number of tasks performed by ICU nurses and the variability in caregiver involvement. The implementation of a nurse mentor can be helpful. Implementation of mentoring (nurse—mentor, family—mentee), based on proven theories, can bring a number of benefits to families and ICU patients and set a new direction for nurses.

## 8. Implication for Future Research

There is a need for further research on interventions that support family involvement in delirium management. The results of existing studies should be confirmed by conducting good-quality RCTs. Future research should clearly define the scope of interventions that support family involvement in delirium management. Studies should evaluate the impact of the interventions used on family outcomes (such as sense of efficacy, anxiety symptoms, depression, and satisfaction with care). Possible measurement tools include the following: Generalized Self-Efficacy Scale (GSES), Self-Efficacy Scales (based on Bandura’s guidelines), General Anxiety Disorder-7 (the GAD-7), the State Trait Anxiety Inventory state (STAIS), Hospital Anxiety and Depression Scale (HADS), and family satisfaction in the intensive care unit 24 (FS-ICU 24). Another area that needs to be explored is how family involvement in delirium management will reduce its incidence among ICU patients. Two tools are recommended for assessing delirium in ICUs: the Confusion Assessment Method for the ICU (CAM-ICU) and the Intensive Care Delirium Screening Checklist (ICDSC).

## Figures and Tables

**Table 1:** Search strategy.

Database	Search Term
Medline	(“Critical Care” [MESH terms] OR Intensive Care Units [MESH Terms]) AND (“Family” [MESH Terms] or “Caregivers” [MESH Terms]) AND (“delirium” [MESH Terms] or “acute confusion” [MESH Terms]) AND (non-pharmacological or psychologically or interventions or education or support or involvement or engagement or participation)
	Limit: Language
	Results: 54
Cinahl	TX (intensive care unit or icu or critical care) AND TX (family or relatives or family members or caregiver) AND TI (delirium or acute confusion or confusion or disorientation) AND TX (interventions or strategies or best practices) AND TX (involvement or participation or engagement)
	Limit: Language, adult, years
	Results: 213
Cochrane Library	TX (intensive care unit or icu or critical care) AND TX (family or relatives or family members or caregiver) AND TX (delirium or acute confusion or confusion or disorientation) AND TX (interventions or strategies or best practices) AND TX (involvement or participation or engagement)
	Limit: Language, years
	Results: 102

**Table 2:** Characteristics of the included studies.

Author, Year	Country	Study Design	Setting	Study Participants	Purpose
Mailhot, T., et al., 2017 [15]	Canada	A randomized pilot study	The surgical ICU, or the surgery unit of the hospital	30 patient/family caregivers	To assess the feasibility, acceptability, and preliminary efficacy of a nursing intervention involving family caregivers (FC) in delirium management following cardiac surgery.
Mailhot, T., et al., 2022 [16]	Canada	Intervention study	Cardiac surgery ICU and the surgery unit	Patients and family caregivers	To develop a nursing intervention (MENTOR_D) to support the involvement of families in delirium management.
Krewulak, KD., 2020 [17]	Canada	Pre-test post-test quasi-experimental	General systems adult ICU	63 family members	To evaluate the effectiveness of an ICU Family Education on Delirium (iFAM-ED) intervention that prepares family members to partner with the ICU care team to detect delirium symptoms and prevent and manage delirium using nonpharmacological strategies.
Hwang JH., et al., 2021 [18]	Korea	Nonequivalent control group pretest-posttest nonsynchronized	ICU	73 participants	To investigate the effects of a multicomponent intervention program for preventing delirium on the incidence of delirium, self-extubation, or self-removal of the catheter, and LOS among elderly patients in ICU.
Wheeler, A., et al., 2023 [19]	USA	A quasi-experimental study	Oncology mixed medical–surgical ICU	31 family caregivers	To determine the feasibility of implementation of a delirium education video for family caregivers (1), to compare knowledge about delirium in caregivers who received the education versus those who did not (2), and to measure anxiety and caregiver satisfaction (3).

**Table 3:** Synthesis of included articles.

Author, Year	Intervention	Outcome Measures	Main Findings		Code
			Caregivers’ Outcomes	Clinical Significance	
Mailhot, T., et al., 2017 [15]	A nurse acted as a mentor who provided information on delirium and guidance to the FC in his or her new role of intervening in delirium management. Examples of nurse-mentor interventions: Give information on appropriate actions to be taken by the FC at the bedside of the patient with delirium; encourage FC to use delirium management interventions at the bedside during delirium; give feedback. Nurse mentoring interventions to family caregivers are presented in the protocol paper of this study (Mailhot et al., 2014 [23]). Starts within 24 h of delirium onset with a total of seven encounters: 30 min pre-bedside phase, 15 min bedside phase, and 15 min post-bedsidephase; and 30 min seventh discharge encounter.	- Number of eligible patients, FC refusal reasons, and length of recruitment (to assess acceptability and feasibility of the study design). - TAPQ, types of FCI provided by more than 50% of FCs when at the bedside (to assess the acceptability and feasibility of the intervention). - DI (to assess outcome of delirium severity). - Medical charting (to assess patient complications during delirium). - SIP (to assess psycho-functional recovery). - STAIS (to assess the FC’s anxiety). - A 14-item Likert scale adapted from Bandura’s guide (to assess self-efficacy). - CAM-ICU (to measure the occurrence of delirium).	✓ Apotential to diminish FC anxiety. ✓ Increased self-efficacy.	✓ Intervention was acceptable and feasible. ✓ A potential to diminish the LoS (postoperative).	Mentoring
Mailhot, T., et al., 2022 [16]	(1) The pre-bedside phase of 30 min (guiding of the family by the nurse mentor to identify and practice appropriate, tailored non-pharmacological management of delirium that could be used during the bedside phase). (2) The bedside phase of 15 min (modeling by the nurse mentor of the non-pharmacological management of delirium and providing the family with enough confidence to tailor this non-pharmacological management). (3) The post-bedside phase of 15 min (the nurse-mentor and family reflect on the bedside phase while offering feedback and preparing for the next visit). This sequence was repeated twice daily for three consecutive days following the onset of delirium.	n/a	✓ Diminished anxiety. * ✓ Increased self-efficacy. *	✓ Diminished delirium severity. * ✓ Diminished complications. * ✓ Diminished LoS. * ✓ Increased recovery. *	Mentoring
Krewulak, KD. 2020 [17]	iFAM-ED intervention consisted of two parts: (1) A six-minute iFAM-ED video module (video module included a definition of delirium, a description of the possible symptoms of delirium, delirium risk factors, symptoms that distinguish delirium from dementia, non-pharmacological treatments to prevent and manage delirium, and how to communicate delirium symptoms to the ICU care team). (2) Previously validated case vignettes of hypothetical ICU patients. After watching the video, family members proceeded to practice detecting mayhem in the case vignettes provided, using two delirium detection tools: FAM-CAM and Sour Seven.	- The CIDKQ-A (immediately after the intervention and two weeks after iFAM-ED). - To assess anxiety: GAD-7.	✓ Family members’ knowledge of delirium significantly improved (in all three aspects: risk factors for delirium, actions to prevent/treat delirium, and the ability to differentiate between symptoms of dementia and delirium). ✓ Knowledge was maintained two weeks after education. ✓ Most family members experienced mild or clinically significant symptoms of anxiety. ✓ No correlation between anxiety symptoms and knowledge of delirium.	Compliance in detecting delirium using the FAM-CAM scale was moderate and significant using Sour Seven.	Education
Hwang, HJ et al., 2021 [18]	Family caregiver education (as one of 8 MIPPDs): -Assess the educational needs and the decision of primary family caregiver. ▪ Educate the family caregiver on the definition, symptoms, etiology, negative effects, and prevention of delirium, and the orientation reinforcement intervention that the family caregiver would take part in. -Schedule the additional visit time. Upon ICU admission: One additional intervention-related visit in addition to the twice-daily visits set by the hospital guidelines.	No data	No data	No data	Education
Wheeler, A. et al., 2023 [19]	During the development of the delirium education video, a partnership was formed with, among others, the hospital’s Patient Education Committee, whose members included patients and their caregivers (in the first step, involved an informal assessment of the family’s needs by interviewing the caregiver of a former ICU patient with delirium). The family caregiver delirium education video was approximately 5 min in length with animations and a voiceover recording on delirium risk factors, signs/symptoms, and ways for family members to provide non-pharmacological interventions. The video integrated a case scenario requiring the viewer to demonstrate the knowledge learned. Complete the study either virtually or in person.	- The CIDKQ questionnaire. - HADS-A. - FS-ICU 24. - Questionnaire on opinion of the educational video (for intervention group).	✓ No significant difference in total knowledge score (further analysis by subgroups showed significantly higher knowledge in the intervention group). ✓ Positive feedback from all intervention participants, with the majority saying they were very satisfied and found the video helpful.	✓ The feasibility of the educational film intervention in both virtual and in-person forms. ✓ Total of 70% of the participants indicated that they used the strategies they learned in the video, and these were as follows: informing the loved one of the date/time/place, and encouraging exercise. ✓ HADS-A i FS-ICU 24 results are not discussed within the scope of this publication.	Partnership/Education

* Anticipated outcomes. FC—family caregiver; FCI—family caregiver interventions; TAPQ—the Treatment Acceptability and Preference Questionnaire; DI—the Delirium Index; SIP—the Sickness Impact Profile; STAIS—the State Trait Anxiety Inventory state; CAM-ICU—The Confusion Assessment Method: Intensive Care Unit; LoS—length of stay; n/a—not applicable; CIDKQ—Caregiver Delirium Knowledge Questionnaire; FAM-CAM—the Family Confusion Assessment Method; iFAM-ED—the Family Education on Delirium; GAD-7—the Generalized Anxiety Scale: Seven; MIPPD—Multi-component Intervention Program for Preventing Delirium; HADSA—Hospital Anxiety and Depression Scale: Anxiety Subsection; FS-ISU 24—the Family Satisfaction Survey for the Intensive Care Unit: 24.

## Data Availability

The authors declare that the data of this research are available from the corresponding author on request.
